# Daptomycin as a possible new treatment option for surgical management of Methicillin-Resistant Staphylococcus aureus sternal wound infection after cardiac surgery

**DOI:** 10.1186/1749-8090-5-57

**Published:** 2010-08-06

**Authors:** Aron F Popov, Jan D Schmitto, Theodor Tirilomis, Christian Bireta, Kasim O Coskun, Suyog A Mokashi, Alexander Emmert, Martin Friedrich, Christoph H Wiese, Friedrich A Schoendube

**Affiliations:** 1 Department of Thoracic and Cardiovascular Surgery, University of Göttingen, Germany; 2 Division of Cardiac Surgery, Department of Surgery, Brigham and Women's Hospital, Harvard Medical School, Boston, MA, USA; 3 Department of Anaesthesiology, Emergency and Intensive Care Medicine, University of Göttingen, Germany

## Abstract

We present a case of a 77-year old female who had undergone a coronary artery bypass grafting with an aortic valve replacement and developed three month later a Methicillin-Resistant Staphylococcus aureus (MRSA) sternal wound infection which was successful treated with Daptomycin combined with vacuum-assisted closure (VAC).

## Introduction

Sternal wound infection is a severe complication in cardiac surgery despite continuing efforts to improve perioperative conditions. This complication is often associated with significant morbidity and mortality rates of up to 45% [1], with prolonged hospitalization [2] and additional surgical procedures, as well as prolonged antibiotic therapy and its inherent high costs [3]. The most common conventional treatments involve surgical revision, open dressing, closed mediastinal irrigation, debridement, complete sternectomy, or reconstruction with omental or muscleflaps [4]. With the increase of MRSA infection, the accompanying antibiotic therapy has received more attention for treatment of sternal wound infections after cardiac surgery.

## Case Report

A 77-year-old female was admitted with coronary artery disease and severe aortic stenosis to the Department of Cardiac Surgery of the University Hospital of Goettingen, Germany in July of 2007. A coronary artery bypass grafting (left anterior descending artery was revascularized by the left internal mammarian artery) and an aortic valve replacement (Cryolife O'Brien^® ^23 mm, biological) were performed. After an uneventful operation and postoperative course, the patient was discharged home. Three month after discharge, at the initial postoperative visit, physical examination revealed an unstable sternum with purulent drainage (MRSA-positive) from the distal portion of the incision. Subsequently, the patient was hospitalized and started on wide broad spectrum antibiotics (Clindamycin and Rifampicin) in combination with local antiseptic washings. She was urgently taken to the operating room for wound debridement. Once the incision was reopened, frank pus was noted. The wound was irrigated and the sternum was realigned. Her general condition recovered and two months after the operation, the patient was discharged home.

One month following this, the patient returned with purulent drainage forming in the distal wound, necessitating hospital readmission with intravenous antibiotics (Vancomycin 500 mg/d, for 10 days). Given the prior presentation of an unstable sternum, we elected to remove three sternal wires. A vacuum-assisted closure (VAC) was placed along with Alginat to promote secondary wound healing. On the 26^th ^postoperative day, the patient was discharged home with instructions to return for clinic three-times-per-week for wound care.

**Figure 1:**
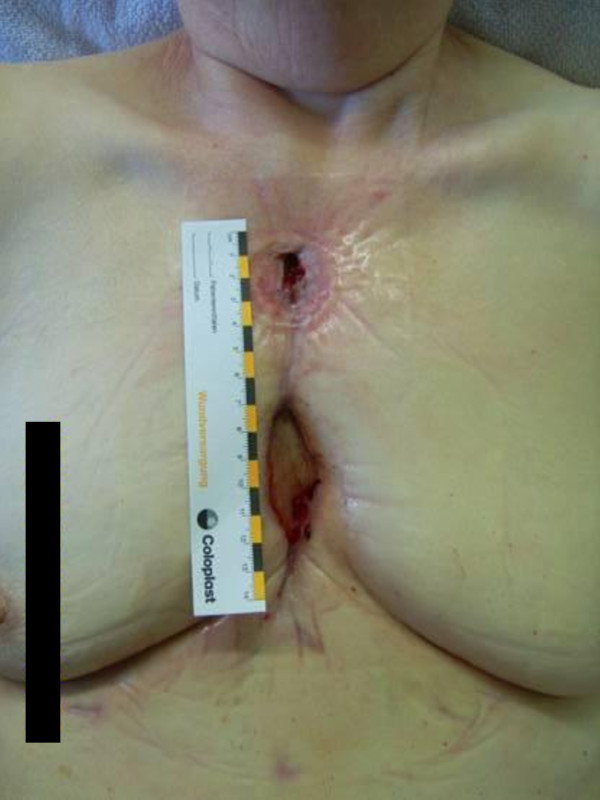
**Postoperative sternal wound infection eight months since the initial surgery**. The remaining two sternal wires were removed. After removing sternal wires, reapplication of VAC therapy was initiated.

One month following this, eight months since the initial surgery, the wound was not fully healed. Although there was some evidence of secondary degree healing, it was felt the patient would benefit from removing the remaining two sternal wires. Therefore, each sternal wire was removed, the wound was widely debridement of infected tissues, and a VAC was placed the entire length of the incision (Figure 1). This resulted in further wound healing and the patient was again discharged home with wound care.

**Figure 2:**
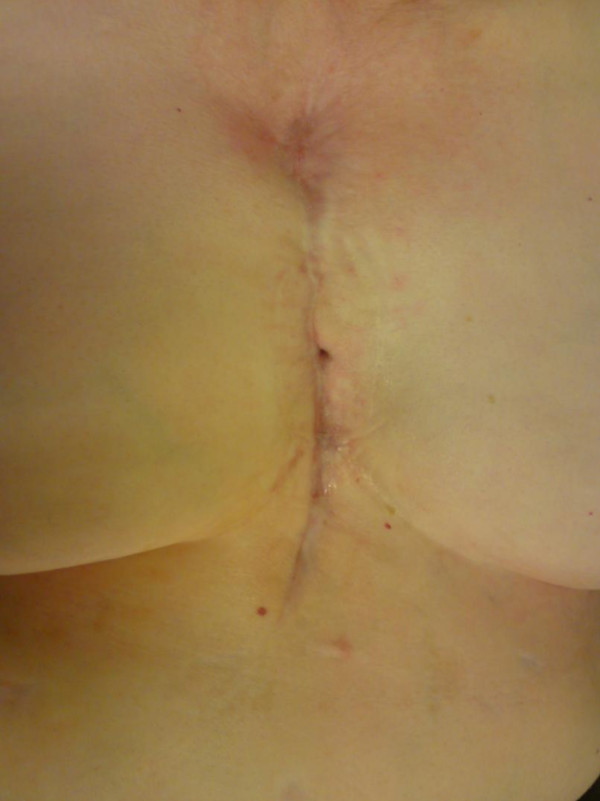
**The wound eventually healed with no residual fistula or infection of MRSA, twenty-one months since the initial surgery**.

In March 2009, twenty months since the initial surgery, the patient presented with yet another sternal wound dehiscence. When the wound was probed, a fistula was noted to the mediastinum. She readmitted to the hospital and brought to the operating room for wound irrigation with VAC placement. Bacterial cultures obtained intraoperatively grew MRSA and the antibiogram presented resistance to several conventional antibiotics but displayed sensitivity to the new antibiotic drug Daptomycin (Cubicin^®^, Novartis Pharma GmbH, Germany). Daptomycin (4 mg/kg/day) was administered and total duration of application was ten days. The wound eventually healed with no residual fistula or infection of MRSA (Figure 2) and she was discharged on the 18th postoperative day. A follow-up visit in May 2010 in our ambulance revealed no indication of bacterial colonization in latest microbiological tests. The patient is free of pain and able to function well in daily life.

## Discussion

The incidence of sternal wound infection after cardiac surgery is reported to be 0.4-5% [5] and Staphylococcus aureus is the most common pathogen isolated from sternal wound infections after cardiac surgery as well as from bacteraemic blood cultures [6]. An increasing trend in antibiotic resistance, with the appearance of progressively more cases of MRSA strain infections have been shown in epidemiological studies [6,3]. Sternal infection with S. aureus is associated with high morbidity and mortality and carries a worse prognosis than that of other aetiologies [7].

Vancomycin remains the reference standard for the treatment of systemic infection caused by methicillin resistant Staphylococcus aureus (MRSA). However, there are many reasons for clinical failure of Vancomycin [8,9], therefore the need for alternative therapies that target MRSA has become apparent. One alternative is Linezolid, because it has been shown that this antibiotic drug in retrospective evaluations of complicated skin and soft-tissue infections (SSTIs) caused by MRSA, compared with Vancomycin, is associated with significantly higher clinical cure rates and reduced lengths of hospitalization [10,11]. Despite the apparent advantages of Linezolid in the treatment of MRSA infections, concerns about safety and costs of therapy often limit its use.

Daptomycin is a lipopeptide drug with bactericidal activity against MRSA in a concentration-dependent manner [12]. The difference between Daptomycin and standard therapy in the treatment of MRSA infections was up to now not statistically significant, however Daptomycin has already been proven to be effective in the treatment of bacteremia and endocarditis caused by MRSA and several case reports about its effectiveness in the field of cardiac surgery exist in the literature [13-17]. Based on these observations, Daptomycin may offer a possible new treatment option for surgical management of MRSA sternal wound infection after cardiac surgery combined with surgical therapy.

In our case the patient was re-submitted to our hospital with generalized colonization and infection with MRSA. Standard therapy concerning antibiotic treatment has failed to eradicate the MRSA, so that we decided for an alternative antimicrobial strategy in the form of Daptomycin application. However, its longterm efficacy in cardiac surgery should be further evaluated in a controlled setting.

## Competing interests

The authors declare that they have no competing interests.

## Authors' contributions

AP and JS had helped with surgical techniques, performed data, analysis, statistics, graphics, and wrote the paper. TT, CB, AE, SM, MF and CW helped with data interpretation and helped to draft the manuscript. FS co-wrote the manuscript and added important comments to the paper. All authors read and approved the final manuscript.

## Consent

Written informed consent was obtained from the patient for publication of this case report and any accompanying images. A copy of the written consent is available for review by the Editor-in-Chief of this journal.

## References

[B1] National Nosocomial Infections Surveillance (NNIS) System Report, data summary from January 1992 to June 2002, issued August 2002 Am J Infect Control 2002 30 458 75 10.1067/mic.2002.130032 12461510

[B2] Kappstein I Schulgen G Fraedrich G Schlosser V Schumacher M Daschner FD Added hospital stay due to wound infections following cardiac surgery Thorac Cardiovasc Surg 1992 40 148 51 10.1055/s-2007-1020134 1412382

[B3] Sharma M Berriel-Cass D Baran J Jr Sternal surgical-site infection following coronary artery bypass graft: prevalence, microbiology, and complications during a 42-month period Infect Control Hosp Epidemiol 2004 25 468 71 10.1086/502423 15242193

[B4] Sjogren J Malmsjo M Gustafsson R Ingemansson R Poststernotomy mediastinitis: a review of conventional surgical treatments, vacuum assisted closure therapy and presentation of the Lund University Hospital mediastinitis algorithm Eur J Cardiothorac Surg 2006 30 898 905 10.1016/j.ejcts.2006.09.020 17056269

[B5] Poncelet AJ Lengele B Delaere B Zech F Glineur D Funken JC El Khoury G Noirhomme P Algorithm for primary closure in sternal wound infection: A single institution 10-year experience Eur J Cardiothorac Surg 2008 33 2 232 38 10.1016/j.ejcts.2007.11.016 18082415

[B6] Fowler VG Jr Kaye KS Simel DL Cabell CH McClachlan D Smith PK Levin S Sexton DJ Reller LB Corey GR Oddone EZ Staphylococcus aureus bacteremia after median sternotomy: clinical utility of blood culture results in the identification of postoperative mediastinitis Circulation 2003 108 73 8 10.1161/01.CIR.0000079105.65762.DB 12821547

[B7] Gårdlund B Bitkover CY Vaage J Postoperative Mediastinitis in cardiac surgery - microbiology and pathogenesis Eur J Cardiothorac Surg 2002 21 825 30 10.1016/S1010-7940(02)00084-2 12062270

[B8] Sakoulas G Moellering RC Jr Eliopoulos GM Adaptation of methicillin-resistant Staphylococcus aureus in the face of vancomycin therapy Clin Infect Dis 2006 42 40 50 10.1086/491713 16323119

[B9] Robert J Bismuth R Jarlier V Decreased susceptibility to glycopeptides in methicillin-resistant Staphylococcus aureus: a 20 year study in a large French teaching hospital, 1983-2002 J Antimicrob Chemother 2006 57 506 10 10.1093/jac/dki486 16410265

[B10] Weigelt J Itani K Stevens D Lau W Dryden M Knirsch C Linezolid CSSTI Study Group Linezolid versus vancomycin in treatment of complicated skin and soft tissue infections Antimicrob Agents Chemother 2005 49 6 2260 6 10.1128/AAC.49.6.2260-2266.2005 15917519 PMC1140485

[B11] Itani KM Weigelt J Li JZ Duttagupta S Linezolid reduces length of stay and duration of intravenous treatment compared with vancomycin for complicated skin and soft tissue infections due to suspected or proven methicillin-resistant Staphylococcus aureus (MRSA) Int J Antimicrob Agents 2005 26 6 442 8 10.1016/j.ijantimicag.2005.09.003 16289514

[B12] Steenbergen JN Alder J Thorne GM Tally FP Daptomycin: a lipopeptide antibiotic for the treatment of serious Gram-positive infections J Antimicrob Chemother 2005 55 283 288 10.1093/jac/dkh546 15705644

[B13] Fowler VG Jr Boucher HW Corey GR Abrutyn E Karchmer AW Rupp ME Levine DP Chambers HF Tally FP Vigliani GA Cabell CH Link AS DeMeyer I Filler SG Zervos M Cook P Parsonnet J Bernstein JM Price CS Forrest GN Fätkenheuer G Gareca M Rehm SJ Brodt HR Tice A Cosgrove SE S. aureus Endocarditis and Bacteremia Study Group Daptomycin versus standard therapy for bacteremia and endocarditis caused by Staphylococcus aureus N Engl J Med 2006 355 7 653 65 10.1056/NEJMoa053783 16914701

[B14] Furuya EY Lowy FD Antimicrobial strategies for the prevention and treatment of cardiovascular infections Curr Opin Pharmacol 2003 3 5 464 9 10.1016/j.coph.2003.05.004 14559089

[B15] Marco F de la Mària CG Armero Y Amat E Soy D Moreno A del Río A Almela M Mestres CA Gatell JM Jiménez de Anta MT Miró JM Hospital Clinic Experimental Endocarditis Study Group Daptomycin is effective in treatment of experimental. endocarditis due to methicillin-resistant and glycopeptide-intermediate Staphylococcus aureus Antimicrob Agents Chemother 2008 52 7 2538 43 10.1128/AAC.00510-07 18426900 PMC2443906

[B16] Schmitto JD Popov AF Sossalla ST Coskun KO Mokashi SA Wintner A Schoendube FA Daptomycin for treatment of methicillin-resistant Staphylococcus epidermidis saphenectomy wound infection after coronary artery bypass graft operation (CABG): a case report J Cardiothorac Surg 2009 4 47 10.1186/1749-8090-4-47 19747387 PMC2753312

[B17] Weis F Beiras-Fernandez A Kaczmarek I Sodian R Vicol C Reichart B Weis M Daptomycin for eradication of a systemic infection with a methicillin-resistant-Staphylococcus aureus in a biventricular assist device recipient Ann Thorac Surg 2007 84 1 269 70 10.1016/j.athoracsur.2007.02.001 17588430

